# The Effect of Heat Stress on Autophagy and Apoptosis of Rumen, Abomasum, Duodenum, Liver and Kidney Cells in Calves

**DOI:** 10.3390/ani9100854

**Published:** 2019-10-22

**Authors:** Ruina Zhai, Xusheng Dong, Lei Feng, Shengli Li, Zhiyong Hu

**Affiliations:** 1 College of Animal Science, Xinjiang Agricultural University, Urumqi 830052, China; 2 Ruminant Nutrition and Physiology Laboratory, College of Animal Science and Technology, Shandong Agricultural University, Taian 271018, China; 3 State Key Laboratory of Animal Nutrition, College of Animal Science and Technology, China Agricultural University, Beijing 100193, China

**Keywords:** autophagy, apoptosis, heat stress, calf

## Abstract

**Simple Summary:**

Heat stress causes significant negative responses in the dairy industry. The objective of this study was to assess the effect of heat stress on the autophagy and apoptosis of the rumen, abomasum, duodenum, liver and kidney in calves. The results showed that heat stress could increase the autophagy and apoptosis of the kidney, duodenum and abomasum. However, heat stress had no effect on the autophagy and apoptosis of the liver. In cows, most studies of autophagy and apoptosis have only focused on mammary remodeling. Our results provide new knowledge regarding autophagy and autophagy in calf heat stress management.

**Abstract:**

The objective of this study was to assess the effect of heat stress on the autophagy and apoptosis of the rumen, abomasum, duodenum, liver and kidney in calves. Two groups of Holstein male calves were selected with similar birth weights and health conditions. Heat stress (HT): Six calves (birth weight 42.2 ± 2.3) were raised from July 15 to August 19. Cooling (CL): Six calves (birth weight 41.5 ± 3.1 kg) were raised from April 10 to May 15. All the calves were euthanized following captive bolt gun stunning at 35 d of age. The expression of protein 1 light chain 3-Ⅱ (LC3-Ⅱ) and caspase3 in the rumen, abomasum, duodenum, liver and kidney were determined by western blotting. In addition, other possible relevant serum biochemical parameters were evaluated. Significant differences were observed in alkaline phosphatase (ALP), albumin (ALB) and glucose (Glu). The results showed that heat stress could increase the autophagy and apoptosis of the kidney, duodenum and abomasum. However, heat stress had no effect on the autophagy and apoptosis of the liver. Additionally, the expression of caspase-3 in the rumen in HT was significantly lower than that in CL. In conclusion, the effects of heat stress on autophagy and apoptosis are organ-specific. The results provide knowledge regarding autophagy and autophagy in calf heat stress management.

## 1. Introduction

Heat stress causes significant negative responses in the dairy industry. Heat stress is caused by excessive temperature conditions that cannot be compensated for by the temperature regulation mechanism of cows. The temperature regulation ability is weaker for young calves than for adult cows, with an upper end of about 29 °C, and heat stress is considered to occur at temperatures greater than 32 °C and 60% humidity [1]. Extensive research has shown the diminution of liver enzyme activities and kidney functions in cows during heat stress [2,3]. In rats and pigs, heat stress apparently promotes intestinal mucosal damage due to reduced intestinal blood flow and tissue hyperthermia [4,5]. The reduced blood flow of the digestive tract is probably harmful to the barrier function integrity of rumen [6].

Autophagy and apoptosis are universal mechanisms that regulate gut homeostasis and reduce digestive tract damage [7,8]. When cells are under stress, autophagy and apoptosis are activated [9]. Autophagy is a specific protein degradation process that has been recognized as an important mechanism for cell survival under stress conditions [10,11]. The protein 1 light chain 3-Ⅱ (LC3-Ⅱ) is a useful marker of autophagic membranes and is essential for the expansion of the early autophagosome during cellular house-keeping and autophagic cell death [12,13]. In elegans, autophagy-related genes are transcriptionally upregulated in response to heat shock [14]. Heat stress also triggers autophagy in different types of cells such as human alveolar basal epithelial cells and rat cardiomyocytes [15]. In vivo apoptosis and autophagy are two forms of physiological and conserved programmed cell death [16]. Apoptosis is characterized by a series of morphological changes, including plasma membrane blebbing, nuclear condensation, and fragmentation, all of which lead to the formation of apoptotic bodies [9]. In general, autophagy is activated first and maintains cell homeostasis [17]. When stress is prolonged or exceeds a threshold, apoptosis is activated [9,18]. In cows, heat stress can induce the apoptosis of granulosa cells, as evidenced by the activation of caspase-3 [19]. Caspase-3 is an executioner caspase which plays an important role in apoptosis [9].

In cows, most studies of autophagy and apoptosis have only focused on mammary remodeling [20,21,22]. Thus far, very little attention has been paid to the role of autophagy and apoptosis in calves. The objective of this study was to assess the effect of heat stress on the autophagy and apoptosis of the rumen, abomasum, duodenum, liver and kidney in calves. We hypothesized that autophagy and autophagy would be stimulated to relieve heat stress. We hope to provide knowledge regarding autophagy and autophagy in calf heat stress management.

## 2. Materials and Methods

The study was conducted at the Shandong high-speed modern dairy farm in Ji Ning, Shandong, China in 2018. Animal care and use were approved and conducted under established standards of the Ethics Committee on animals of Shandong Agricultural University (SDAUA-2018-012). 

Two groups of Holstein male calves were selected with similar birth weights and health conditions. Heat stress (HT): Six calves (birth weight 42.2 ± 2.3) were raised from July 15 to August 19. Cooling (CL): Six calves (birth weight 41.5 ± 3.1 kg) were raised from April 10 to May 15. Calves were individually housed in 1.5 × 3.4 m pens inside a naturally ventilated barn with free-choice water and solid feed. The HT calves were housed in the same pens and were only provided with shade. The relative humidity and air temperature of each pen were recorded at 7 days before euthanasia. The temperature humidity index (THI) of the HT and CL groups were calculated as described previously [23]. In the HT group, the average THI was 85.08, whereas the average the THI was 63.49 in the CL group. All the calves were provided with 4 L of colostrum within 2 hours from birth. From the next day, calves were fed 6 L of whole milk once daily until being euthanized. The ingredient and nutrient composition of the calf starter is given in Table 1.

The rectal temperature and respiratory rate of each calf were recorded 3 times per day. At 35 ± 2 d of age, fifteen milliliters of blood were collected from the caudal vein using 20 ml syringes. The samples were collected in the procoagulant tube, centrifuged at 1000× *g* for 15 min, and then the serum was collected in a 1.5 ml Eppendorf tube and stored at −20 °C. All the calves were euthanized following captive bolt gun stunning at 35 ± 2 d of age. After the opening of the body cavity, the samples of the rumen, abomasum, duodenum (entire wall from 6 cm distal to the pylorus), liver and left kidney were washed with normal saline. Then, these samples were frozen in liquid nitrogen and stored at −80 °C until western blotting was performed.

Briefly, the tissue samples blocks were washed with PBS (Solarbio, P1020-500ml, Beijing, China), cut into small pieces, homogenized in PBS at 4 °C using a Servicebio KZ-Ⅱ homogenizer, kept on ice for 0.5 h, oscillated to ensure complete tissue cracking every 5 min, and then centrifuged (3000 × g, 10 min, 4 °C). Protein concentration was detected by a bicinchoninic acid (BCA) Protein Assay Kit (G2026, Servicebio, Wuhan, China). A Laemmli sample buffer (Bio-Rad, 1610737, Shanghai, China) was used to dilute the sample and then boiled for 5 min. Immunoblotting was performed as previously described [24,25]. The LC3 (Sigma-Aldrich, L8918, Shanghai, China), caspase-3 (Sigma-Aldrich, C8487, Shanghai, China) and β-actin (Sigma-Aldrich, A2066, Shanghai, China) antibodies and appropriate secondary antibodies (Servicebio, GB23303, Wuhan, China) were applied according to the manufacturer’s guidelines. The chemiluminescence of the bands of interest was detected with a digital G: Box imager (Syngene, Frederick, MD, USA). Image J software (National Institutes of Health, Bethesda, MD, USA) was used to quantify the band density. Alanine aminotransferase (ALT), aspartate aminotransferase (AST), alkaline phosphatase (ALP), total protein (TP), albumin (ALB), glucose (Glu) and total cholesterol (TCHO) of the serum were determined with an automatic biochemical analyzer (Type 7020, Hitachi, Tokyo, Japan). 

The data were analyzed with a completely randomized design using a one-way ANOVA of SAS 8.2 (SAS Institute Inc., Cary, NC, USA). The individual calf was considered as the experimental unit. The means were compared using Duncan’s multiple range test. Significance was declared at *p* < 0.05.

## 3. Results

In the HT group, the average THI was 85.08, whereas the average THI was 63.49 in the CL group. It could also be seen that the rectal temperature (39.36 ± 0.26 vs. 38.31 ± 0.19 °C, respectively; *p* < 0.01) and respiratory rate (55.17 ± 3.49 vs. 34.17 ± 2.48 breaths per minute; respectively; *p* < 0.01) of the HT calves were significantly higher than the CL calves. Those data indicated that the HT calves were exposed to heat stress, while the CL calves were not subjected to heat stress.

**Figure 1:**
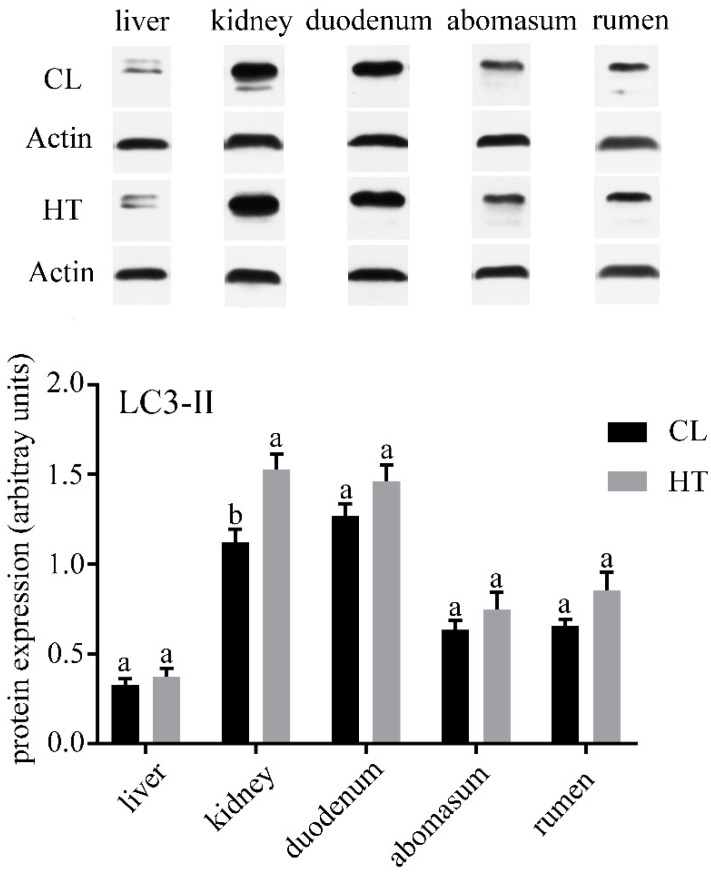
Effects of the temperature humidity index (THI) on the microtubule-associated protein 1 light chain 3-Ⅱ (LC3-Ⅱ) expression of the liver, kidney, duodenum, abomasum and rumen in the HT and CL calves. Treatment was as follows: (1) HT: Calves were fed from July 15 to August 19; (2) CL: Calves were fed from April 10 to May 15. Insets depict representative blots. Values represent means ± standard deviation Response from statistical result, *p* < 0.05. β-Actin was used to normalize the expression of target proteins. The letters below the bar graph indicate different organs. Different letters above the bar indicate differences between different groups (*p* < 0.05).

**Figure 2:**
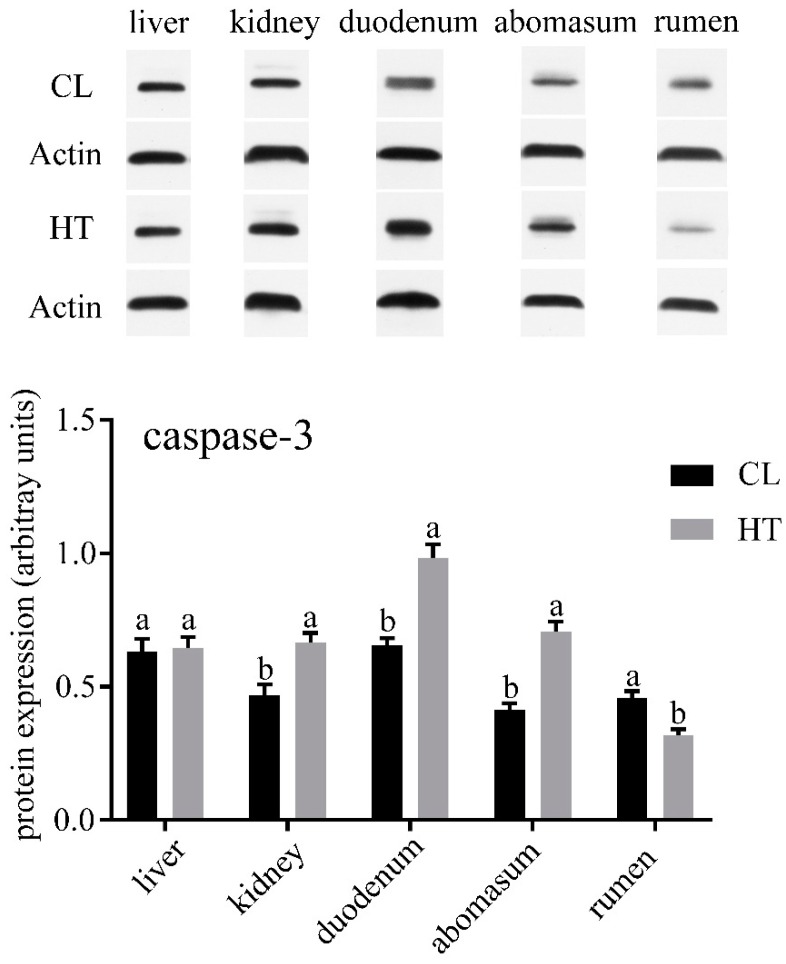
Effects of the THI on the caspase3 expression of the liver, kidney, duodenum, abomasum and rumen in the HT and CL calves. Treatment was as follows: (1) HT: Calves were fed from July 15 to August 19; (2) CL: Calves were fed from April 10 to May 15. Insets depict representative blots. Values represent means ± standard deviation. Response from statistical result, *p* < 0.05. β-Actin was used to normalize the expression of target proteins. The letters below the bar graph indicate different organs. Different letters above the bar indicate differences between different groups (*p* < 0.05).

No differences were observed in the concentrations of ALT, AST, TP and TCHO in plasma of two groups (Table 2). ALP and ALB in the CL group were significantly higher than that in the HT group (*p* < 0.05), while the CL calves had a lower amount of serum Glu (*p* < 0.05). Compared with the CL calves, the HT calves had a lower expression of LC3-Ⅱ in the kidney (*p* < 0.05) and tended to have a lower expression in the duodenum, abomasum and rumen (Figure 1). The expressions of caspase-3 in the kidney, duodenum and abomasum were elevated in the HT calves relative to the CL calves (*p* < 0.01). No significant differences were found in caspase-3 expression of the liver between the two groups (Figure 2). Interestingly, the expression of caspase-3 in the rumen in the HT group was significantly lower than that of the CT group.

## 4. Discussion

In our study, the THI, rectal temperature and respiratory rate indicated that the HT calves were exposed to significant environmental heat stress, while the CL calves did not suffer from heat stress. The current study found that the HT group had a higher concentration of Glu than the CL group. This result agrees with an earlier study, which showed that Glu tended to have a higher concentration in an HT group [26]. However, a previous study found that pancreatic insulin response to Glu stimulation and the concentration of insulin in calves were not affected by the heat stress [27,28]. It seems possible that the high concentration of serum Glu was connected to the impaired cell metabolism and osmolarity caused by heat stress without the change of insulin. It has been suggested that ALP plays a vital role in bone mineralization and hepatobiliary diseases [29,30]. The low concentration of ALP in the HT calves may impact the development of bone and hepatobiliary. ALB may be able to regulate oncotic pressure and modulate inflammatory or immunological responses [31]. In our study, the concentration of serum ALB was lower in the HT calves than the CL calves. Our result agrees with a previous study, indicating that heat stress could decrease the concentration of serum ALB, which perhaps, in turn, could impact oncotic pressure [31].

We analyzed the expression of LC3-II and caspase-3 of the HT and CL calves to assess the effect of the THI on autophagy and apoptosis in calves. In our study, we found that different organs have different levels of autophagy and apoptosis. All the organs in our study except liver were affected by heat stress. In the duodenum and abomasum, especially the kidney, the level of autophagy and apoptosis were increased by heat stress. A previous study found that heat stress could induce autophagy in hepatocellular carcinoma [32]. In the skeletal muscle of *Sus scrofa*, the markers of autophagosome formation and autophagic activation were increased by heat stress [33]. These results were similar to those of our study. The enhanced autophagy may be a self-protection mechanism of organs under heat stress.

Hyperosmolarity, which is one of the consequences of heat stress, could activate several mediator systems that may cause renal injury [34]. Calves extensively sweating results in a serious loss of water and salt, which could lead to an increase of urine-specific gravity and osmolarity [34]. In our study, the high concentration of Glu in the HT calves may have been due to the increase of osmotic pressure. Additionally, the activity of aldose reductase, which can convert Glu into sorbitol and is increased by hyper osmolarity [35]. Sorbitol can protect kidney cells from the hyperosmotic environments under the conditions of plasma hyperosmolarity and dehydration [36,37]. Previous studies have found that hyperosmotic stress could induce apoptosis and suppressed mammalian target of rapamycin complex 1 (mTORC1) which could inhibit autophagy [38,39]. However, sorbitol dehydrogenase could convert sorbitol into fructose. In the small intestine, the metabolism of fructose is associated with local inflammation and increased intestinal permeability [40,41]. These physiological duodenal changes probably caused the high level of apoptosis in the duodenum seen in our study.

One interesting finding was that the level of autophagy and apoptosis in the liver were not affected by heat stress. Autophagy, which is a response to stressful conditions of the liver, could eliminate damaged mitochondria and accumulated lipid droplets in liver [42]. Both autophagy and apoptosis play important roles in liver injury [42]. It seems possible that the liver has a strong regulatory ability to reduce the damage caused by heat stress. Additionally, the apoptosis level was decreased by heat stress in the rumen. It seems that the effects of heat stress on autophagy and apoptosis are organ-specific. Further work is required to evaluate the effect of osmotic pressure on autophagy and apoptosis. In addition, further study should focus on the effects of heat stress on the liver.

## 5. Conclusions

In conclusion, heat stress could increase the level of autophagy and apoptosis of the kidney, duodenum and abomasum. However, heat stress has no effect on the autophagy and apoptosis of the liver. Heat stress decreased serum ALP and ALB and increased Glu concentration. In conclusion, the effects of heat stress on autophagy and apoptosis are organ-specific. These results provide knowledge regarding autophagy and autophagy in calf heat stress management.

## Figures and Tables

**Table 1:** Ingredient and nutrient composition of the experimental calves’ starter.

Items	Content (% of DM)
Ingredients, % of DM	
Corn grain	48
Wheat bran	12.6
Soybean meal	18.8
Extruded soybean	7
Corn gluten meal	9
Salt	0.55
Calcium carbonate	2
Dicalcium phosphate	1.15
Vitamin and trace mineral premix ^1^	0.9
Nutrients % of DM	
DM, %	89.3
CP, %	22.13
Crude fat, %	4.32
NDF, %	17.14
ADF, %	6.62
Ca, %	1.07
P, %	0.56
ME, Mcal/kg	2.83

DM: dry matter; CP: crude protein; NDF: neutral detergent fiber; ADF: acid detergent fiber; ME: metabolizable energy.^1^ Premix contained (mg/kg): vitamin A, 4,035; vitamin D, 1,740; vitamin E, 39; Fe, 18; Zn, 37; Cu, 10.6; Mn, 15.3; Co, 0.12; I, 0.47; and Se, 0.35.

**Table 2:** Serum concentrations of alanine aminotransferase (ALT), aspartate aminotransferase (AST), alkaline phosphatase (ALP), total protein (TP), albumin (ALB), glucose (Glu) and total cholesterol (TCHO) of the calves in the CL (cooling) and HT (heat stress) groups.

Item	CL	HT	*p*-Value	SEM
ALT, U/L	17.00	13.00	0.063	1.094
AST, U/L	44.78	44.98	0.959	1.908
ALP, U/L	274.99	191.16	0.082	24.229
TPB, g/L	59.48	62.79	0.490	2.261
ALB, g/L	24.36	22.75	0.007	0.334
Glu, mmol/L	4.97	6.29	0.014	0.291
TCHO, mmol/L	2.44	2.28	0.211	0.0631

## References

[1] Moore D.A., Duprau J.L., Wenz J.R. (2012). Short communication: Effects of dairy calf hutch elevation on heat reduction, carbon dioxide concentration, air circulation, and respiratory rates. J. Dairy Sci..

[2] Nardone A., Ronchi B., Lacetera N., Ranieri M.S., Bernabucci U. (2010). Effects of climate changes on animal production and sustainability of livestock systems. Livest. Sci..

[3] Schneider P.L., Beede D.K., Wilcox C.J. (1988). Nycterohemeral patterns of acid-base status, mineral concentrations and digestive function of lactating cows in natural or chamber heat stress environments. J. Anim. Sci..

[4] Lambert G.P., Gisolfi C.V., Berg D.J., Moseley P.L., Oberley L.W., Kregel K.C. (2002). Selected contribution: Hyperthermia-induced intestinal permeability and the role of oxidative and nitrosative stress. J. Appl. Physiol. (1985).

[5] Pearce S.C., Sanz-Fernandez M.V., Hollis J.H., Baumgard L.H., Gabler N.K. (2014). Short-term exposure to heat stress attenuates appetite and intestinal integrity in growing pigs. J. Anim. Sci..

[6] Kadzere C.T., Murphy M.R., Silanikove N., Maltz E. (2002). Heat stress in lactating dairy cows: A review. Livest. Prod. Sci..

[7] Negroni A., Cucchiara S., Stronati L. (2015). Apoptosis, Necrosis, and Necroptosis in the Gut and Intestinal Homeostasis. Mediat. Inflamm..

[8] Randall-Demllo S., Chieppa M., Eri R. (2013). Intestinal epithelium and autophagy: Partners in gut homeostasis. Front. Immunol..

[9] Marino G., Niso-Santano M., Baehrecke E.H., Kroemer G. (2014). Self-consumption: The interplay of autophagy and apoptosis. Nat. Rev. Mol. Cell Biol..

[10] Sakiyama T., Musch M.W., Ropeleski M.J., Tsubouchi H., Chang E.B. (2009). Glutamine increases autophagy under Basal and stressed conditions in intestinal epithelial cells. Gastroenterology.

[11] Huang H., Li X., Zhuang Y., Li N., Zhu X., Hu J., Ben J., Yang Q., Bai H., Chen Q. (2014). Class A scavenger receptor activation inhibits endoplasmic reticulum stress-induced autophagy in macrophage. J. Biomed. Res..

[12] Klionsky D.J., Abdalla F.C., Abeliovich H., Abraham R.T., Acevedo-Arozena A., Adeli K., Agholme L., Agnello M., Agostinis P., Aguirre-Ghiso J.A. (2012). Guidelines for the use and interpretation of assays for monitoring autophagy. Autophagy.

[13] Tanida I., Ueno T., Kominami E. (2004). LC3 conjugation system in mammalian autophagy. Int. J. Biochem. Cell Biol..

[14] Kumsta C., Chang J.T., Schmalz J., Hansen M. (2017). Hormetic heat stress and HSF-1 induce autophagy to improve survival and proteostasis in C. elegans. Nat. Commun..

[15] Dokladny K., Zuhl M.N., Mandell M., Bhattacharya D., Schneider S., Deretic V., Moseley P.L. (2013). Regulatory coordination between two major intracellular homeostatic systems: Heat shock response and autophagy. J. Biol. Chem..

[16] Yi H.Y., Yang W.Y., Wu W.M., Li X.X., Deng X.J., Li Q.R., Cao Y., Zhong Y.J., Huang Y.D. (2018). BmCalpains are involved in autophagy and apoptosis during metamorphosis and after starvation in Bombyx mori. Insect Sci..

[17] Liu G., Pei F., Yang F., Li L., Amin A.D., Liu S., Buchan J.R., Cho W.C. (2017). Role of Autophagy and Apoptosis in Non-Small-Cell Lung Cancer. Int. J. Mol. Sci..

[18] Kroemer G., Marino G., Levine B. (2010). Autophagy and the integrated stress response. Mol. Cell.

[19] Li L., Wu J., Luo M., Sun Y., Wang G. (2016). The effect of heat stress on gene expression, synthesis of steroids, and apoptosis in bovine granulosa cells. Cell Stress Chaperones.

[20] Wohlgemuth S.E., Ramirez-Lee Y., Tao S., Monteiro A.P.A., Ahmed B.M., Dahl G.E. (2016). Short communication: Effect of heat stress on markers of autophagy in the mammary gland during the dry period. J. Dairy Sci..

[21] Zarzynska J., Motyl T. (2008). Apoptosis and autophagy in involuting bovine mammary gland. J. Physiol. Pharmacol. Off. J. Polish Physiol. Soc..

[22] Teplova I., Lozy F., Price S., Singh S., Barnard N., Cardiff R.D., Birge R.B., Karantza V. (2013). ATG proteins mediate efferocytosis and suppress inflammation in mammary involution. Autophagy.

[23] Tao S., Bubolz J.W., do Amaral B.C., Thompson I.M., Hayen M.J., Johnson S.E., Dahl G.E. (2011). Effect of heat stress during the dry period on mammary gland development. J. Dairy Sci..

[24] Nestal de Moraes G., Carvalho E., Maia R.C., Sternberg C. (2011). Immunodetection of caspase-3 by Western blot using glutaraldehyde. Anal. Biochem..

[25] Wohlgemuth S.E., Seo A.Y., Marzetti E., Lees H.A., Leeuwenburgh C. (2010). Skeletal muscle autophagy and apoptosis during aging: Effects of calorie restriction and life-long exercise. Exp. Gerontol..

[26] Guo J.R., Monteiro A.P.A., Weng X.S., Ahmed B.M., Laporta J., Hayen M.J., Dahl G.E., Bernard J.K., Tao S. (2016). Short communication: Effect of maternal heat stress in late gestation on blood hormones and metabolites of newborn calves. J. Dairy Sci..

[27] Tao S., Monteiro A.P., Hayen M.J., Dahl G.E. (2014). Short communication: Maternal heat stress during the dry period alters postnatal whole-body insulin response of calves. J. Dairy Sci..

[28] Min L., Cheng J.B., Shi B.L., Yang H.J., Zheng N., Wang J.Q. (2015). Effects of heat stress on serum insulin, adipokines, AMP-activated protein kinase, and heat shock signal molecules in dairy cows. J. Zhejiang Univ. Sci. B.

[29] Sharma U., Pal D., Prasad R. (2014). Alkaline phosphatase: An overview. Indian J. Clin. Biochem..

[30] Zierk J., Arzideh F., Haeckel R., Cario H., Fruhwald M.C., Gross H.J., Gscheidmeier T., Hoffmann R., Krebs A., Lichtinghagen R. (2017). Pediatric reference intervals for alkaline phosphatase. Clin. Chem. Lab. Med..

[31] Baldassarre M., Naldi M., Domenicali M., Volo S., Pietra M., Dondi F., Caraceni P., Peli A. (2017). Simple and rapid LC-MS method for the determination of circulating albumin microheterogeneity in veal calves exposed to heat stress. J. Pharm. Biomed. Anal..

[32] Jiang J., Chen S., Li K., Zhang C., Tan Y., Deng Q., Chai Y., Wang X., Chen G., Feng K. (2019). Targeting autophagy enhances heat stress-induced apoptosis via the ATP-AMPK-mTOR axis for hepatocellular carcinoma. Int. J. Hyperthermia.

[33] Ganesan S., Pearce S.C., Gabler N.K., Baumgard L.H., Rhoads R.P., Selsby J.T. (2018). Short-term heat stress results in increased apoptotic signaling and autophagy in oxidative skeletal muscle in Sus scrofa. J. Therm. Biol..

[34] Johnson R.J., Rodriguez-Iturbe B., Roncal-Jimenez C., Lanaspa M.A., Ishimoto T., Nakagawa T., Correa-Rotter R., Wesseling C., Bankir L., Sanchez-Lozada L.G. (2014). Hyperosmolarity drives hypertension and CKD--water and salt revisited. Nat. Rev. Nephrol..

[35] Bardoux P., Bichet D.G., Martin H., Gallois Y., Marre M., Arthus M.F., Lonergan M., Ruel N., Bouby N., Bankir L. (2003). Vasopressin increases urinary albumin excretion in rats and humans: Involvement of V2 receptors and the renin-angiotensin system. Nephrol. Dial. Transplant..

[36] Burg M.B. (1995). Molecular basis of osmotic regulation. Am. J. Physiol..

[37] Schmolke M., Schilling A., Keiditsch E., Guder W.G. (1996). Intrarenal distribution of organic osmolytes in human kidney. Eur. J. Clin. Chem. Clin. Biochem. J. Forum Eur. Clin. Chem. Soc..

[38] Burg M.B., Ferraris J.D., Dmitrieva N.I. (2007). Cellular response to hyperosmotic stresses. Physiol. Rev..

[39] Su K.H., Dai C. (2017). mTORC1 senses stresses: Coupling stress to proteostasis. Bioessays.

[40] Ishimoto T., Lanaspa M.A., Le M.T., Garcia G.E., Diggle C.P., Maclean P.S., Jackman M.R., Asipu A., Roncal-Jimenez C.A., Kosugi T. (2012). Opposing effects of fructokinase C and A isoforms on fructose-induced metabolic syndrome in mice. Proc. Natl. Acad. Sci. USA.

[41] Johnson R.J., Rivard C., Lanaspa M.A., Otabachian-Smith S., Ishimoto T., Cicerchi C., Cheeke P.R., Macintosh B., Hess T. (2013). Fructokinase, Fructans, Intestinal Permeability, and Metabolic Syndrome: An Equine Connection?. J. Equine Vet. Sci..

[42] Wang K. (2015). Autophagy and apoptosis in liver injury. Cell Cycle.

